# Percutaneous radiofrequency lesions adjacent to the dorsal root ganglion alleviate spasticity and pain in children with cerebral palsy: pilot study in 17 patients

**DOI:** 10.1186/1471-2377-10-52

**Published:** 2010-06-22

**Authors:** Georges F Vles, Johan S Vles, Maarten van Kleef, Jan van Zundert, Heleen M Staal, Wim E Weber, Lodewijk W van Rhijn, Dan Soudant, H Kerr Graham, Anton J de Louw

**Affiliations:** 1 Neurology, Maastricht University Medical Centre, Maastricht, the Netherlands; 2 Anesthesiology, Maastricht University Medical Centre, Maastricht, the Netherlands; 3 Orthopaedic surgery, Maastricht University Medical Centre, Maastricht, the Netherlands; 4 Orthopaedic Surgery, Royal Children's Hospital, Melbourne, Australia; 5 Epilepsy Centre Kempenhaeghe, Heeze, the Netherlands

## Abstract

**Background:**

Cerebral palsy (CP) may cause severe spasticity, requiring neurosurgical procedures. The most common neurosurgical procedures are continuous infusion of intrathecal baclofen and selective dorsal rhizotomy. Both are invasive and complex procedures. We hypothesized that a percutaneous radiofrequency lesion of the dorsal root ganglion (RF-DRG) could be a simple and safe alternative treatment. We undertook a pilot study to test this hypothesis.

**Methods:**

We performed an RF-DRG procedure in 17 consecutive CP patients with severe hip flexor/adductor spasms accompanied by pain or care-giving difficulties. Six children were systematically evaluated at baseline, and 1 month and 6 months after treatment by means of the Modified Ashworth Scale (MAS), Gross Motor Function Measure (GMFM) and a self-made caregiver's questionnaire. Eleven subsequent children were evaluated using a Visual Analogue Scale (VAS) for spasticity, pain and ease of care.

**Results:**

A total of 19 RF-DRG treatments were performed in 17 patients. We found a small improvement in muscle tone measured by MAS, but no effect on the GMFM scale. Despite this, the caregivers of these six treated children unanimously stated that the quality of life of their children had indeed improved after the RF-DRG. In the subsequent 11 children we found improvements in all VAS scores, in a range comparable to the conventional treatment options.

**Conclusion:**

RF-DRG is a promising new treatment option for severe spasticity in CP patients, and its definitive effectiveness remains to be defined in a randomised controlled trial.

## Background

Cerebral palsy (CP) is a central nervous system deficit resulting from a non-progressive lesion in the developing brain. Although these brain lesions are static, the movement disorders that arise are not unchanging and are characterised by atypical muscle tone, posture and movement [1]. The spastic motor type is the most common form of CP and its conventional therapeutic management may include splinting/casting, physiotherapy, occupational therapy, oral spasmolytics and anti-dystonic drugs, Botulinum Toxin-A (BTX-A) injections, orthopaedic procedures, and neurosurgical procedures. The most common neurosurgical procedures are continuous infusion of intrathecal Baclofen (ITB) and selective dorsal rhizotomy (SDR). Since its first description by Foerster in 1913, SDR has been modified by various researchers and has become a standard neurosurgical procedure to treat spasticity in CP patients [2-5]. However, as multiple-level laminectomies at the L1-S1 level are often required, the procedure is invasive and can lead to complications as transient urinary incontinence, chronic low back pain and spinal deformity [6-9]. Considerable cooperation of the patient is required to successfully complete the postoperative rehabilitation program.

An alternative for the SDR is a radiofrequency lesion of the dorsal root ganglion (RF-DRG), which has been used to treat chronic pain for over 30 years [10]. The RF-DRG is a simple and safe treatment with little side effects [11-13]. In the 1980's several authors reported that the same procedure might be used to treat adult patients with spasticity [14,15]. More recently, we were able to show that an RF-DRG alleviates hip flexor spasms in 2 CP patients [16]. CP patients can also suffer from severe pain through spasticity, bone deformities or joint subluxation, especially hip displacement and dislocation [17,18], which may lead, through an enhanced processing of afferent information within the spinal cord, to secondary pain [19].

We thus hypothesized that an RF-DRG may alleviate spasticity and pain in CP patients, and we tested this hypothesis in a pilot study of 17 patients.

## Methods

Children with spasticity from CP were seen by a multi-disciplinary spasticity management team (child neurologist, orthopaedic surgeon, and physician assistant child neurology) in the Maastricht University Medical Centre(JV, LvR, HS, DS). Individual treatment goals were determined after a careful assessment of the aetiology, functional ability and associated impairments as a result of the spasticity. The RF-DRG treatment was considered in children with severe hip flexor/adductor spasms accompanied by pain or care giving difficulties. The procedure was approved by the Institutional Review Board of the Maastricht University Medical Centre, according to Dutch governmental regulations and informed consent from the caregivers was obtained for each patient.

A total of 17 children were treated with RF-DRG (tables 1 and 2). The severity of CP was based on the Gross Motor Function Classification System (GMFCS) for CP [20,21]. Hip(sub)luxation was classified according to the morphological hip classification system proposed by Robin et al [22].

**Table 1:** Summary of 6 patients (Group A) treated with RF-DRG and evaluated systematically

	m/f	Age (yr)	Aetiology	GMFCS score	RF-DRG-left	RF-DRG-right	Indication			Improvement 4w			Side-Effects 4w	Improvement 6m			Side-Effects 6m	Hip morphology	
							spast	pain	care	spast	pain	care		spast	pain	care		Left	Right
																			
1	f	6	Asphyxia	V	L1-2	L1-2	▪		▪	+			no	+			no	IV	III
2	f	12	Trauma	V	L1-4	L1-4	▪	▪	▪	+	+	+	no	+	+	+	no	III	I
3	f	16	Premature	V	-	L1-3		▪	▪		+	+	no		+	+	no	II	V
4	f	11	Asphyxia	V	L2-4	L2-4	▪			+			no	+			no	II	IV
5	m	7	Premature	V	L1-2	-		▪			-		yes		+		no	V	III
6	f	14	Asphyxia	V	-	L1-3	▪	▪	▪	+	+	+	no	+	+	+	no	III	V

m: male; f: female; yr: years; GMFCS: Gross Motor Function Classification System; spast: spasticity

▪: indication

Improvement: +/-: summary of improvement/worsening measured by the self-made questionnaire

4w: 4 weeks; 6m: 6 months

**Table 2:** Summary of 11 patients (Group B) treated with RF-DRG and evaluated by VAS.

	m/f	Age (yr)	SCPE	GMFCS Score	RF-DRG-left	RF-DRG-right	Indication			VAS pre	Improvement			VAS post	Δ-VAS	Side-effects	Hip morphology	
							spast	pain	care		spast	pain	care				left	right
1	f	18	2	V	L1-3	-		▪		8		+		2	6	no	III	V
2	f	22	2	V	L1-4	-	▪	▪		10	+	+		1.5	8.5	no	ns	ns
3	f	10	2	V	L1-2	L1-2	▪	▪		10	+			8	2	no	V	III
4	m	16	2	V	L1-3	-		▪		7		+		3	4	no	II	V
5	m	16	2	V	L1-4	-	▪	▪		8.2		+		1.6	6.6	no	IV	V
6	m	7	2	V	-	L1-3	▪			ns				ns		yes	0	III
7	m	5	2	V	L1-3	L1-3	▪		▪	ns			+	ns		no	IV	IV
8	f	11	2	V	L1-2	-		▪		6		+		2	4	no	*	II
9	f	10	2	V	L1-2	-	▪	▪	▪	8		+	+	5.5	2.5	no	I	I
	f	12	2	V	L1-2	-	▪	▪	▪	8		+	+	5	3	no	I	I
	f	14	2	V	L1-2	-	▪	▪	▪	10				10	0	no	I	I
10	m	5	2	V	L1-4	L1-4	▪		▪	ns	+			ns		no	II	V
11	f	16	2	V	L1-4	L1-4	▪	▪	▪	8.7	+	+	+	3.1	5.6	no	ns	ns

m: male; f: female; yr: years; SCPE: surveillance for cerebral palsy in Europe classification: (1) unilateral; (2) bilateral

GMFCS: Gross Motor Function Classification System * Osteomyelitis; ns: not scored;

▪: indication

+: domain in which the improvement measured by VAS was noted

Six children (group A) were systematically evaluated at baseline, and 1 month and 6 months after treatment (table 1, table 3). In this group all assessments were performed by a physical therapist. The Modified Ashworth Scale (MAS) was used for the assessment of changes in muscle tone [23]. In the MAS the muscle tone is scored on a 6 point scale, in which "0" represents no hypertonia or no increase of muscle tone and "4" represents severe hypertonia and severe stiffness of the extremities. In each patient hip flexion and adduction, knee flexion and extension and dorsal and plantar flexion in the ankle were tested bilaterally. Functional improvement was assessed using the Gross Motor Function Measure (GMFM), a widely used scale consisting of 5 different locomotor domains to evaluate treatment of spasticity in children with CP [24-26]. Furthermore, we developed a more extensive questionnaire for caregivers. Besides items on pain, several items of daily activities of life, like dressing, undressing and bathing, were investigated. Data of the 6 systematically evaluated children were statistically analyzed using a students T-test and a Wilcoxon signed rank test. A p value of < 0.05 was considered to be statistically significant. All data are represented as means and standard error of means (SEM).

**Table 3:** Modified Ashworth Scale scores of the first 6 patients: pre-operative, post-operative 4 weeks and 6 months.

		Hipflexion		Hip adduction		Knee flexion		Knee extension		Ankle dorsal flexion		Ankle plantar flexion	
Pt		L	R	L	R	L	R	L	R	L	R	L	R
1	Pre	1+	1+	1	2	1	2	0	0	3	3	0	0
	Post4w	1	1	0	0	1	1	0	0	2	2	0	0
	Post6m	1	0	1	1	1	0	0	0	3	2	0	0
2	Pre	0	0	1	3	0	0	1	1	3	3	0	0
	Post4w	0	0	1	1	0	0	1	1	1	1	0	0
	Post6m	0	0	1	1	0	0	0	0	1	1	0	0
3	Pre	0	1	1+	2	1	1	2	2	3	3	0	1
	Post4w	0	1+	1+	1	1+	1	2	1+	2	2	0	0
	Post6m	2	1	2	1	2	1	0	0	2	2	0	0
4	Pre	0	0	1	1	1+	1+	3	3	0	0	1	1
	Post4w	0	0	0	0	2	2	0	0	1	0	0	0
	Post6m	1	1	0	0	2	2	0	0	1	1	0	0
5	Pre	2	0	0	1	0	1+	2	2	2	2	1+	1+
	Post4w	1	1	0	1	1	2	1	4	1	1	2	3
	Post6m	2	0	0	0	2	4	0	0	2	3	0	0
6	Pre	3	3	4	4	4	4	4	4	4	3	4	3
	Post4w	0	0	1	0	3	1	2	3	2	1+	2	2
	Post6m	3	3	4	3	3	3	0	0	4	4	4	4

Mean	Pre	1	0.83	1.33	2.17	1.17	1.5	2	2	2.5	2.33	1	1
	Post4w	0.33	0.5	0.75	0.5	1.33	1.17	1	1.5	1.5	1.17	1.67	0.83
	Post6m	1.5	0.83	1.33	1.0	1.67	1.67	0.17	0	2.17	2.17	0.67	0.67

**Figure 1:**
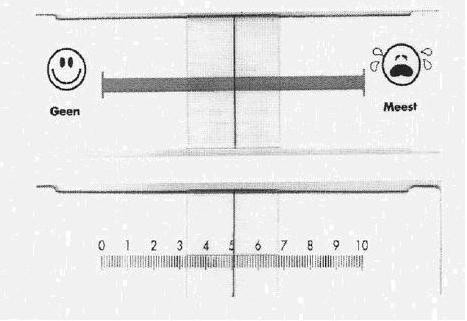
**The VAS device**. Score 0/"geen": Very satisfied. Score 10/"meest": Very dissatisfied. Questions: 1. What overall score would you give your child when considering pain, ease of care and spasticity? 2. In which domain(s) did you notice this improvement: pain, ease of care, spasticity? (See table 2)

The following 11 children (group B) were evaluated at 6 weeks and 3 months using a Visual Analogue Scale (VAS), which was used to measure the severity of the individually formulated problems. We changed the evaluation methods, as we were not able to detect the kind of change that the caregivers unanimously did notice. Furthermore, we learned from literature that the MAS and the GMFM are not indicated to measure changes in these severely handicapped children [27-29]. Instead we used a VAS, which is a valid pain-rating instrument [30]. The VAS used in pain assessment is a straight 10 cm horizontal line with anchor points of no pain (score 0) and unbearable pain (score 10). For our study we changed the anchor points into very satisfied (score 0) and very dissatisfied (score 10) to use the VAS uniformly for individually defined problems [[31,32], figure 1]. A VAS score for spasticity, pain, and ease of care was given by the caregivers, since the majority of the CP patients were severely mentally handicapped. Usually the patient and his/her caregiver(s) are the best judges of the severity of the impairments accompanying spasticity, as they are the only people who can assess its impact on the daily life of the patient [33]. Caregivers of children with profound impairments note changes in function far more accurately than staff workers [34].

### RF-DRG procedure

All patients were treated in our outpatient clinic, and RF-DRG was performed as described [12,13,35]. Under general anaesthesia, the patients were placed in prone position on an operating table. The level to be treated was based on clinical symptoms; afterwards stimulation was used to verify these levels.

The procedure was performed in tunnel vision, a technique for entering the electrode in the direct vision of the X-rays. Therefore, the C-arm (Ziehm electronics) was adjusted in such a way that the X-rays ran parallel to the end plates of the relevant level. Thereafter, the C-arm was rotated until the processus spinosus projected over the contralateral facet column. With the C-arm in this projection, the injection point was found by projecting a metal ruler over the lateral part of the foramen intervertebrale.

**Figure 2:**
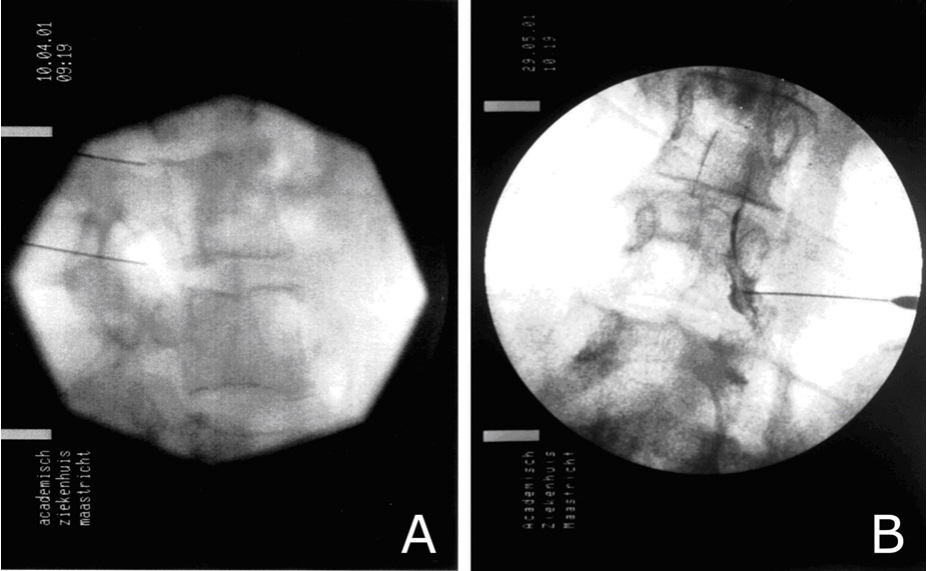
**Photomicrograph showing the position of an RF electrode a: lateral view and b: anterior- posterior view after injection of Omnipaque**.

A 10-cm long, 22-G needle SMK-5 mm tip was inserted locally in the direction of the X-rays. Thereafter, the direction was corrected in such a way that the needle was being projected as a point on the screen. The direction of the radiation beam was now modified to a profile (lateral) view, and the needle was inserted until the point was located in the craniodorsal part of the foramen intervertebrale (Figure 2a).

In an AP view, the course of a small amount of contrast agent was followed with "real-time imaging"; it should spread out laterocaudally along the spinal nerve (Figure 2b). The stylet was removed and exchanged for the radio frequent probe. After checking the impedance (an indicator for the type of tissue next to the cannula tip), electrical stimulation was started at a rate of 2 Hz and the corresponding muscles were observed for contractions. At 2 Hz stimulation motor contractions should be observed in the area of the relevant muscles at a threshold between 0.4-0.8 Volts. 1 ml local anaesthetic solution (bupivacain 0.5%) was injected and radio frequent current was then led through the electrode in order to increase the temperature to 67°C for 60 seconds. Total operating time, including anaesthesia, was about 45 minutes. All patients were discharged from the clinic the same day after full recovery from the anaesthesia.

## Results

A total of 19 RF-DRG treatments were performed in 17 patients. One patient received three interventions, as the effects gradually wore off. A summary of the clinical data, including treated levels and outcome measures, are given in table 1 (group A: 6 patients) and table 2 (group B: 11 patients). In 2 patients a transient increase in pain after the RF-DRG procedure was noted, and 1 patient was successfully treated with Gabapentine for 4 weeks. No further side effects (e.g. dysaesthesia or excessive weakness in the treated limbs) were reported. No significant differences were observed between the different lumbar levels for both the impedance and the stimulation threshold.

### Group A

In the first six systematically evaluated children (table 1) an improvement in muscle tone after RF-DRG was detected on both the short (4 weeks) and long (6 months) term using the MAS. Especially the right hip adductors showed improvement on the MAS which went from 2.2 +/- 0.5 before RF-DRG to 0.5 +/- 0.2 four weeks after RF-DRG and 1.0 +/- 0.4 six months after RF-DRG (table 3). Due to the small sample size, no comment on the lack of significance of a null result can be given.

Using the GMFM no improvement was observed after RF-DRG treatment. At baseline a mean total score including all 5 domains of 17.2% +/- 6 was found. After 4 weeks the score was 17.3% +/- 7 and after 6 months the score was 16.2% +/- 6.

Using the caregiver's questionnaire, an improvement in ease and quality of care and pain was detected. Although statistically not significant, in four patients pain in the lower extremities decreased, at both 4 weeks and 6 months. In one patient there was no change in pain and in another patient pain increased at 4 weeks but decreased at 6 months when compared to the baseline.

### Group B

Because of the abovementioned results we switched to a VAS to evaluate the initiated treatment in the subsequent patients. This score was available in 8 out of the 11 patients. The missing values are due to the fact that some caregivers did not feel comfortable judging their own child in a numerical way. During outpatient follow up, the evaluation of the VAS scores showed improvement in most of the defined goals in most children (table 2). Most parents reported that the positive effects of the RF-DRG lasted at least 6-9 months.

In one patient (no. 9), the VAS score showed no change after the third procedure. Her flexor spasms were then successfully treated with Botulinum Toxin-A injections in the iliopsoas and adductor muscles (Dysport, Ipsen).

## Discussion

To test our hypothesis that RF-DRG may be a serious treatment option for severe spasticity in children with CP, we undertook a pilot study in 17 patients. Our primary treatment goal was improvement of well-being and ease of care of CP patients with severe hip flexor/adductor spasms and pain. Our first 6 consecutively treated patients (Group A) were evaluated at baseline, 1 month after treatment and 6 months after treatment with the GMFM and the MAS. We found a small improvement in muscle tone by the MAS, but no effect on the GMFM scale. Despite this, the caregivers of these six treated children unanimously stated that the quality of life of their children had indeed improved after the RF-DRG (data not shown, summary represented by +/- in table 1). This disagreement between the MAS and the judgement of the caregivers highlights the difficulties measuring change in these severely handicapped children. The MAS as a method for the evaluation of the treatment of spasticity in children with CP has been disputed before, although in adult populations the reliability of the MAS has been demonstrated [27]. In a study in children with moderate to severe spasticity, a wide variability in test-retest results was reported for the MAS [28]. The assumption that the MAS purely measures spasticity is not entirely right. The MAS measures a broader set of neural and musculoskeletal factors of non-velocity-dependent hypertonia in addition to spasticity itself [29].

Furthermore, we added up the MAS scores of different muscles to produce a summed Ashworth score, in order to compare with most previous studies. However, this might be methodologically incorrect because the Ashworth score is an ordinal level measure [36].

For the next 11 patients we thus changed our evaluating system: instead of the MAS and the GMFM, we asked the caregivers to give a VAS score for spasticity, pain and ease of care at different time points. Using this outcome measure we were able to show a significant improvement in spasticity, pain and ease of care after an RF-DRG procedure, lasting up to 9 months. In two patients we found a transient increase in pain, for which 1 patient received Gabapentine for 4 weeks. This is a known transient procedure related event [37]. Although this was not a controlled study, the amount of improvement in VAS scores is in the same range as in conventional treatments, e.g. intrathecal baclofen (ITB) and SDR [31,38]. RF-DRG is thus a promising new treatment option for severe spasticity in CP patients, and its definitive effectiveness remains to be defined in a randomised controlled trial. It is less invasive and has probably less side effects than ITB and SDR, but its main disadvantage in this patient population is, of course, the temporary character of its effects. In this regard RF-DRG resembles BTX-A treatment, but its main advantage over this therapeutic option is its strong pain-reducing effect. Theoretically, repetitive RF-DRG's may lead to dysesthesias and causalgias, but we have not seen this in the one patient we treated three times [35]. To prevent this possible side effect, more definitive effects of RF-DRG might perhaps be achieved with higher radiofrequency currents and temperatures during the procedure.

Our pilot study does highlight the methodological problem of evaluating treatment effects in children with CP. As we were not able to conduct an extensive battery of clinical tests in our pilot study, we had to select a few outcome measures. Using the MAS and the GMFM, we were not able to detect any beneficial effects, which is in line with other studies [33]. We had to resort to a VAS to find improvements in the condition of treated patients. In retrospect we should also have included the Pediatric Evaluation of Disability Inventory (PEDI), which is a generic standardised instrument used by the multidisciplinary team for evaluating functional performance, programme monitoring, documentation of functional development and clinical decision-making [31,38,39].

The exact mechanism of action of an RF-DRG lesion remains unknown. We recently reported an increase of proliferation inside the dorsal root ganglion after RF-DRG adjacent to the ganglion without signs of neural tissue damage (e.g. necrosis) inside the ganglion [40]. For years, the only mode of action of a radiofrequency lesion was thought to be through nerve damage due to thermocoagulation [41]. Recent experiments with pulsed radiofrequency treatment (a high-frequency current delivered in bursts of 20 ms followed by a silent period of 480 ms, during which the generated heat is washed out) suggest that thermocoagulation is not the only mode of action [41,42]. A late and temperature-independent increase in the expression of the immediate early *c-fos *gene within the rat spinal cord was found after exposure of the cervical dorsal root ganglion to continuous radiofrequency (67°C) and pulsed radiofrequency current [43]. In one model of spasticity, the locomotor abnormalities are thought to be the result of hyperexcitability of spinal interneurons involved in the spinal stretch reflex [44]. Reduction of spinal input through de-afferentation is then the basic mechanism of RF-DRG.

## Conclusion

Our pilot study on RF-DRG in 17 CP patients with severe hip flexor/adductor spasms and pain shows that it may improve spasticity, pain, and ease of care, with a duration of up to 9 months. As the amount of improvement is in the same range as in conventional treatments, RF-DRG is thus a promising new treatment option for severe spasticity in CP patients, and its definitive effectiveness remains to be defined in a randomised controlled trial. The main advantages are the less invasive character of RF-DRG compared to the SDR with the potential benefit of a shorter hospitalisation period, and its clear pain-reducing effect.

## Competing interests

The authors declare that they have no competing interests.

## Authors' contributions

JV originated the idea, is a member of the multi-disciplinary working group and together with LR and HS selected the patients. HS and LR are also members of the multi-disciplinary working group and together with GV they scored hip classification (inter-observer agreement with each other). MK and JZ performed the procedure. DS is the supervisor of the multi-disciplinary working group and provided the date for GV which he analysed together with AL. GV wrote the article under supervision of AL, WW and JV. MK en JV participated in writing the methods-sections of the article. AL, HS, KG and WW have revised the manuscript critically for important intellectual content. JV is the final supervisor of the study and manuscript.

All authors read and approved the final manuscript.

## Pre-publication history

The pre-publication history for this paper can be accessed here:

http://www.biomedcentral.com/1471-2377/10/52/prepub
